# Low-Dose Nitric Oxide as Targeted Anti-biofilm Adjunctive Therapy to Treat Chronic *Pseudomonas aeruginosa* Infection in Cystic Fibrosis

**DOI:** 10.1016/j.ymthe.2017.06.021

**Published:** 2017-07-24

**Authors:** Robert P. Howlin, Katrina Cathie, Luanne Hall-Stoodley, Victoria Cornelius, Caroline Duignan, Raymond N. Allan, Bernadette O. Fernandez, Nicolas Barraud, Ken D. Bruce, Johanna Jefferies, Michael Kelso, Staffan Kjelleberg, Scott A. Rice, Geraint B. Rogers, Sandra Pink, Caroline Smith, Priya S. Sukhtankar, Rami Salib, Julian Legg, Mary Carroll, Thomas Daniels, Martin Feelisch, Paul Stoodley, Stuart C. Clarke, Gary Connett, Saul N. Faust, Jeremy S. Webb

**Affiliations:** 1 NIHR Southampton Respiratory Biomedical Research Centre, Southampton SO16 6YD, UK; 2 University Hospital Southampton NHS Foundation Trust, Southampton SO16, 6YD, UK; 3 Centre for Biological Sciences, University of Southampton, Southampton SO17 1BJ, UK; 4 Institute for Life Sciences, University of Southampton, Southampton SO17 1BJ, UK; 5 Faculty of Medicine, Clinical and Experimental Sciences, University of Southampton, Southampton SO17 1BJ, UK; 6 Microbial Infection and Immunity, The Ohio State University College of Medicine, Columbus, OH 43210-2210, USA; 7 Southampton NIHR Wellcome Trust Clinical Research Facility, Southampton SO16 6YD, UK; 8 Imperial College London School of Public Health, London SW7 2AZ, UK; 9 Centre for Marine Bio-Innovation and School of Biotechnology and Biomolecular Sciences, University of New South Wales, Sydney, NSW 2052, Australia; 10 Kings College London Institute of Pharmaceutical Science, London WC2R 2LS, UK; 11 Public Health England, Southampton SO17 1BJ, UK; 12 Illawarra Health and Medical Research Institute and School of Chemistry, University of Wollongong, Wollongong, NSW 2522, Australia; 13 Singapore Centre on Environmental Life Sciences Engineering and Nanyang Technological University, School of Biological Sciences, Singapore 637551, Singapore; 14 Infection and Immunity Theme, South Australia Health and Medical Research Institute, North Terrace, Adelaide, SA 5000, Australia; 15 Flinders University School of Medicine, Bedford Park, Adelaide, SA 5042, Australia; 16 National Centre for Advanced Tribology at Southampton, Faculty of Engineering, University of Southampton, Southampton SO17 1BJ, UK

**Keywords:** *Pseudomonas aeruginosa*, cystic fibrosis, nitric oxide

## Abstract

Despite aggressive antibiotic therapy, bronchopulmonary colonization by *Pseudomonas aeruginosa* causes persistent morbidity and mortality in cystic fibrosis (CF). Chronic *P. aeruginosa* infection in the CF lung is associated with structured, antibiotic-tolerant bacterial aggregates known as biofilms. We have demonstrated the effects of non-bactericidal, low-dose nitric oxide (NO), a signaling molecule that induces biofilm dispersal, as a novel adjunctive therapy for *P. aeruginosa* biofilm infection in CF in an ex vivo model and a proof-of-concept double-blind clinical trial. Submicromolar NO concentrations alone caused disruption of biofilms within ex vivo CF sputum and a statistically significant decrease in ex vivo biofilm tolerance to tobramycin and tobramycin combined with ceftazidime. In the 12-patient randomized clinical trial, 10 ppm NO inhalation caused significant reduction in *P. aeruginosa* biofilm aggregates compared with placebo across 7 days of treatment. Our results suggest a benefit of using low-dose NO as adjunctive therapy to enhance the efficacy of antibiotics used to treat acute *P. aeruginosa* exacerbations in CF. Strategies to induce the disruption of biofilms have the potential to overcome biofilm-associated antibiotic tolerance in CF and other biofilm-related diseases.

## Teaser

This paper reports the first example of targeted anti-biofilm therapy in human disease. We have demonstrated that using low-dose nitric oxide as a non-bactericidal signaling molecule to induce biofilm dispersal may be useful as a novel adjunctive therapy to treat chronic pseudomonal biofilm infection in cystic fibrosis.

## Introduction

Cystic fibrosis (CF) is the most common lethal, hereditary disease in Caucasian populations, with a United Kingdom and United States incidence of approximately 1 in 2,500 live births and an estimated worldwide prevalence of 70,000.^1^, ^2^ Long-term morbidity and mortality are primarily associated with the effects of chronic *Pseudomonas aeruginosa* lung infection and the persistence of *P. aeruginosa* biofilms.^3^, ^4^ Bacteria in biofilms are enclosed in a self-produced biopolymeric matrix and display up to 1,000-fold higher tolerance to antibiotic challenge than their single-cell, planktonic (free living) counterparts.^5^ Biofilms also exhibit resistance to phagocytosis and other components of the host’s innate and adaptive immune system.^6^ Biofilm survival mechanisms include impedance of antibiotic diffusion through the biofilm matrix,^7^ altered growth or metabolic rates of bacterial subpopulations within the biofilm,^8^, ^9^ and physiological,^8^ biochemical,^10^ and genetic^11^, ^12^ changes. In addition, sub-inhibitory levels of aminoglycoside antibiotics can enhance biofilm formation under laboratory conditions.^13^ Biofilms can be firmly attached to tissue but can also exist in the protected phenotype as aggregates in the mucus of the CF lung.^14^ Biofilms are extremely difficult to eradicate using conventional therapeutic regimes.^15^ New approaches targeting chronic biofilm infections are needed for more effective treatment of *P. aeruginosa* in CF and other biofilm-related diseases.^16^

**Figure 1:**
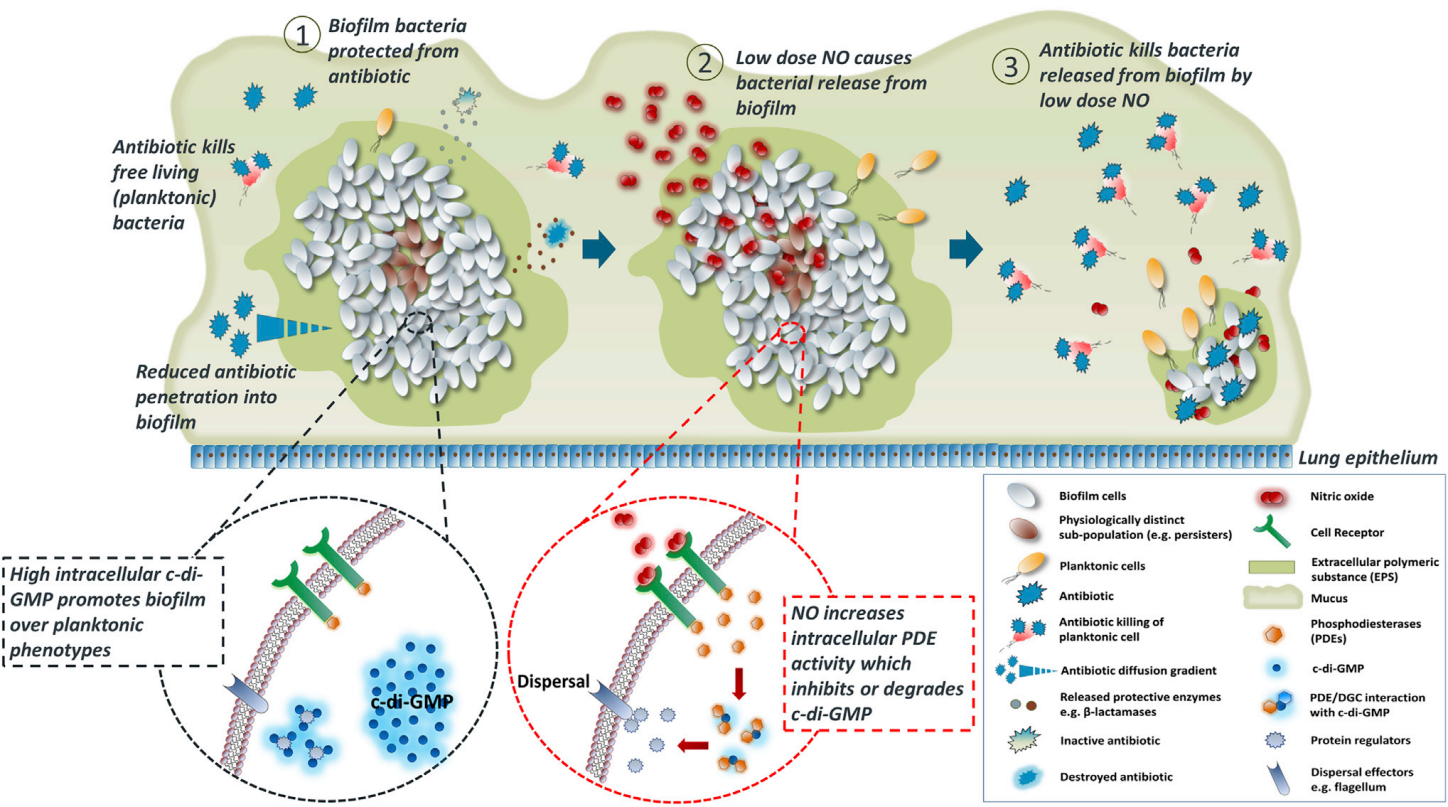
Role of NO in Disrupting Antibiotic Tolerance Mechanisms Associated with the Biofilm Structure (1) Biofilm tolerance mechanisms include reduced antibiotic diffusion, release of protective enzymes capable of destroying or inactivating antibiotics in the biofilm matrix, and formation of physiologically distinct bacterial subpopulations (e.g., persister cells) resulting from nutrient and oxygen gradients. (2) Low-dose NO diffuses into the biofilm and interacts with cell receptors that upregulate cellular phosphodiesterases (PDEs), which accelerate c-di-GMP degradation. This prevents c-di-GMP from interacting with proteins at the transcriptional, translational, or post-translational level and leads to cell surface and physiological changes associated with dispersal and motility (red circle inset). (3) Dispersal is accompanied by reversion of the bacteria to a planktonic phenotype that renders them more susceptible to antibiotic-mediated killing.^18^, ^19^

In vivo, bacteria often transition between planktonic and biofilm lifestyles. Given the correct environmental cues, biofilm bacteria undergo coordinated dispersal and reversion to the planktonic form.^17^ We identified a role for the signaling molecule nitric oxide (NO) in the dispersal of *P. aeruginosa* biofilms^18^, ^19^ (Figure 1). At nanomolar concentrations, NO mediates dispersal by increasing bacterial phosphodiesterase activity with a consequent reduction of the intracellular second messenger and biofilm regulator cyclic-di-guanosine monophosphate (c-di-GMP).^18^, ^19^ Here we report the effects of non-bactericidal, low-dose NO on clinical pseudomonal biofilms ex vivo in the laboratory using conventional and molecular microbiological methods. We have also extended our laboratory findings to a proof-of-concept clinical trial in humans, demonstrating a significant direct effect on pseudomonal biofilm load in CF patients treated with NO gas plus conventional intravenous antibiotic therapy compared with intravenous antibiotics alone.

## Results

### Nitric Oxide Induces *P. aeruginosa* Biofilm Dispersal in Human CF Sputum Samples

**Figure 2:**
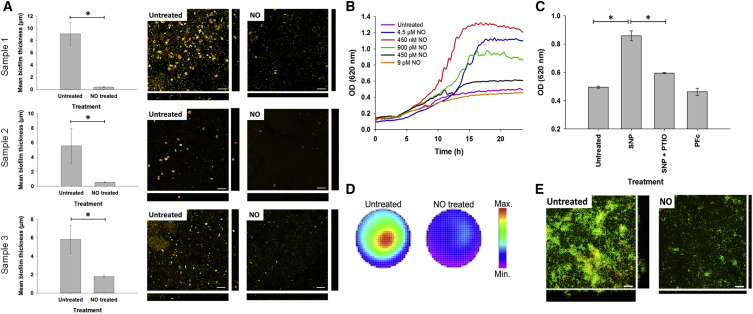
NO Disperses *P. aeruginosa* Biofilms In Vitro and In Cystic Fibrosis Sputum (A) Direct measurement of NO-induced *P. aeruginosa* biofilm dispersal in expectorated CF sputum samples. Image analysis shows a significant reduction in mean *P. aeruginosa* biofilm thickness following treatment of CF sputum samples from three different patients (samples 1, 2, and 3) with 450 nM NO compared with buffer alone (untreated) (*p = 0.02, representing a statistically significant difference between data medians). *P. aeruginosa* was identified using fluorescence in situ hybridization (FISH) with both a Cy3-labeled *P. aeruginosa*-specific 16S rRNA probe (green) and a Cy5-labeled eubacterial 16S probe (red). Confocal laser-scanning microscopy (CLSM) images show a reduction of *P. aeruginosa* (yellow because of hybridization with both probes) in biofilms. Images show horizontal xy (top-down view) sections, and flanking images show vertical z (side view) CSLM sections of untreated (left) and NO-treated (right) CF sputum samples. Scale bars, 25 μm. (B) Nitric oxide (NO) disperses in vitro biofilms grown from biofilm-forming *P. aeruginosa* CF clinical isolates. Dispersal of biofilm bacteria into the planktonic phase (measured by mean OD of overlying planktonic suspensions) following treatment of a clinical *P. aeruginosa* biofilm isolate with low-dose NO (9 pM to 4.5 μM) derived from the spontaneous NO donor SNP. The depicted recordings are from a single isolate and representative of qualitatively identical data from 12 *P. aeruginosa* isolates studied. (C) Biofilm dispersal is NO-dependent. Mean OD measurements of planktonic bacteria following 15-hr treatment of *P. aeruginosa* biofilms with SNP alone, SNP in the presence of the NO scavenger PTIO, or with potassium ferricyanide alone (PFc). *p = 0.02, representing a statistically significant difference between data medians). Data are from three experiments with four wells per experiment. (D) Dispersal causes biofilm detachment from the base of tissue culture plate wells, indicated by loss of fluorescence after NO treatment, compared with untreated controls. Residual biofilms were fluorescently labeled with the nucleic acid probe Syto9. The scale indicates fluorescence intensity, with red corresponding to the highest concentration of surface-attached *P. aeruginosa* and blue-purple corresponding to the fewest remaining attached bacteria. (E) NO induces dispersal of *P. aeruginosa* biofilms in vitro. Representative CSLM images indicate reduced *P. aeruginosa* in biofilms from CF isolates following NO treatment compared with untreated biofilms. Each image shows horizontal xy (top-down view) CLSM sections (square), and flanking images show vertical z (side view) CLSM sections after staining biofilms with the BacLight Live (green)/Dead (red) kit. Scale bars, 25 μm.

NO-induced dispersal of *P. aeruginosa* biofilms was measured directly in expectorated sputum samples from five CF patients using fluorescence in situ hybridization (FISH). A significant reduction in mean biofilm thickness was observed upon treatment with 450 nM NO (generated from the spontaneous NO donor sodium nitroprusside [SNP]; Materials and Methods), and *P. aeruginosa* biofilm microcolonies (aggregates typically ∼15 μm in diameter) were visibly disrupted by NO in five of five patient samples. Figure 2A shows representative experiments from three different patients: sample 1 (p = 0.003), sample 2 (p = 0.029), and sample 3 (p = 0.029).

### Nitric Oxide-Mediated Dispersal of CF *P. aeruginosa* Isolates Occurs within 5–10 hr

Addition of NO (in the form of the NO donor SNP) to 12 biofilm-forming *P. aeruginosa* clinical isolates from CF sputum samples consistently caused dispersal, leading to steep increases in the optical density (OD; turbidity) of planktonic bacterial suspensions overlying biofilms after 5 hr (Figure 2B). The increase in OD correlated with a decrease in biofilm biomass from surfaces of plate wells, as determined by fluorometric measurements and confocal microscopy, confirming the dispersal effect of NO (Figures 2D and 2E). Biofilm dispersal was confirmed to be NO-specific using the NO scavenger 2-phenyl-4,4,5,5-tetramethyl-imidazoline-1-oxyl-3-oxide (PTIO), which reduced the dispersal of *P. aeruginosa* induced by SNP (p = 0.002) to levels similar to the control treatment (Figure 2C). Treatment of biofilms with potassium ferricyanide (as a control for NO-independent breakdown products of SNP) had no dispersal effect compared with untreated biofilms (p = 0.394; Figure 2C). Dispersal was observed at NO concentrations as low as 450 pM, peaking at 450 nM (Figure 2B), with higher concentrations of NO (4.5 μM) showing reduced efficacy for biofilm dispersal (Figure 2B). NO at a concentration of 450 nM dispersed all 12 biofilm-forming CF clinical isolates tested.

### Nitric Oxide Potentiates Antibiotics to Disrupt and Kill Clinical *P. aeruginosa* Biofilms

**Figure 3:**
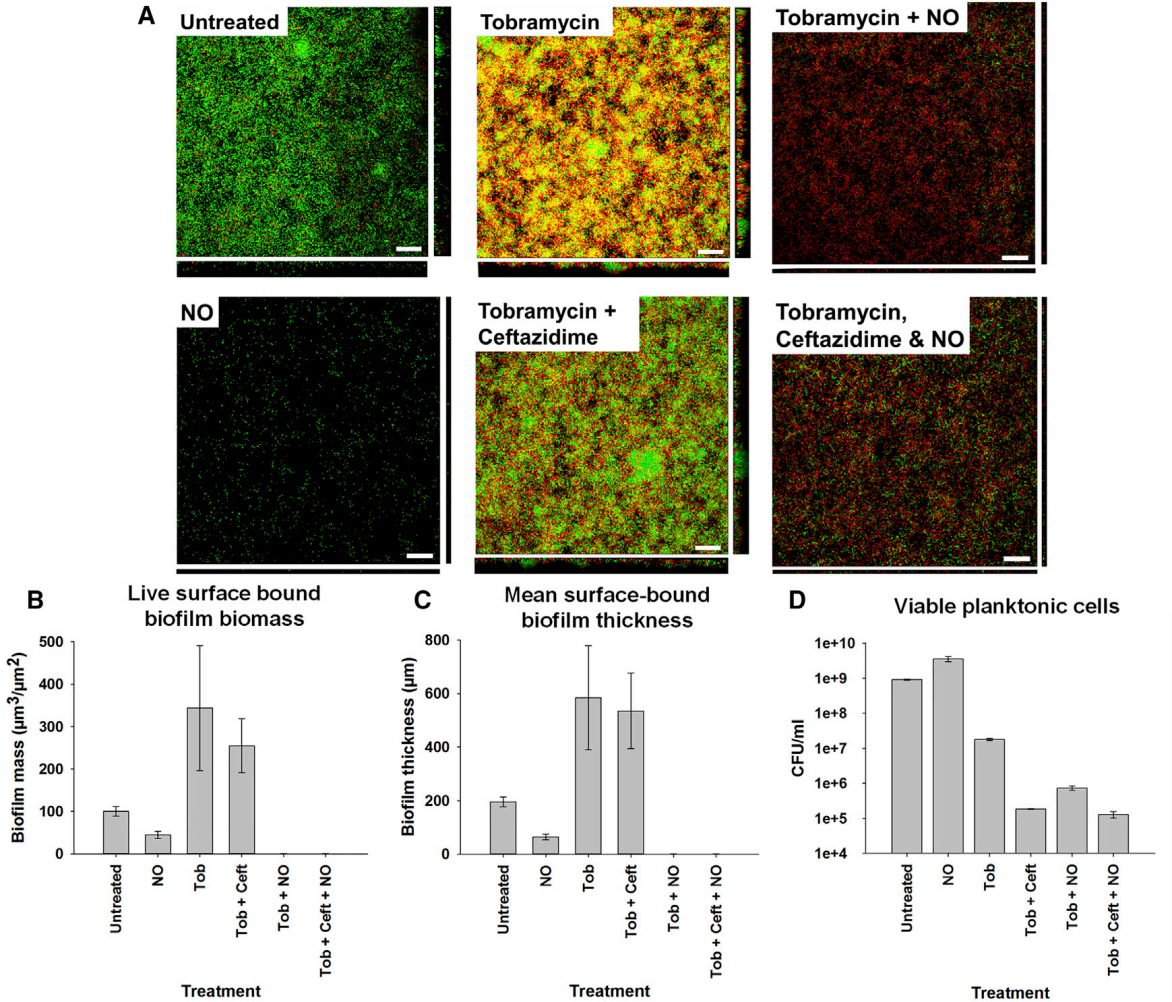
Antibiotic Efficacy against *P. aeruginosa* Clinical Isolate Biofilms Is Enhanced in the Presence of Low-Dose NO (A) Representative CLSM images showing surface-attached *P. aeruginosa* following treatment with buffer alone (untreated), NO alone, MBC antibiotics (5 μg ml^−1^ tobramycin with or without 5 μg ml^−1^ ceftazadime), or antibiotics combined with NO. Images show horizontal xy (top-down view) sections, and flanking images show vertical z (side view) CSLM sections. Biofilms were stained with the BacLight Live (green)/Dead (red) kit to indicate viable cells. Scale bars, 25 μm. (B and C) Image analysis of CLSM images of residual *P. aeruginosa* biofilms with adjunctive NO shows a reduction in mean total biomass (B) and biofilm thickness (C) following treatment with antibiotics (tobramycin alone and tobramycin [Tob]/ceftazidime [Ceft] combined), indicating that NO treatment reduces the amount of remaining biofilm bacteria (error bars represent SEM of five different microscopic fields). An increase in biofilm biomass and biofilm thickness is shown following tobramycin treatment alone (biofilm biomass: 243% increase compared with control, p = 0.028, B; mean biofilm thickness: 199% increase compared with control, p = 0.065, C) and the tobramycin/ceftazidime combination (biofilm biomass: 155% increase compared with control, p = 0.04, B; mean biofilm thickness: 174% increase compared with control, p = 0.04, C). (D) Viable *P. aeruginosa* in the dispersed population (planktonic suspension), determined by colony-forming unit (CFU) counts of *P. aeruginosa* following antibiotic treatment of biofilms with or without NO, indicate that combined NO treatment leads to killing of the bacteria released from the biofilm.

*P. aeruginosa* clinical isolate biofilms treated with the antibiotic tobramycin alone or with tobramycin/ceftazidime combinations were compared with biofilms treated with NO alone, a combination of NO and tobramycin, or a combination of NO, tobramycin, and ceftazidime (Figure 3). Remarkably, the biomass and thickness of the *P. aeruginosa* biofilm increased substantially following antibiotic treatments in the absence of NO. Compared with untreated biofilms, an increase in biofilm biomass and biofilm thickness was observed following tobramycin treatment alone (biofilm biomass: 243% increase compared to control, p = 0.028, Figure 3B; mean biofilm thickness: 199% increase compared with control, p = 0.065, Figure 3C) and the tobramycin/ceftazidime combination (biofilm biomass: 155% increase compared with control, p = 0.04, Figure 3B; mean biofilm thickness: 174% increase compared with control, p = 0.04, Figure 3C). Viability staining demonstrated that predominantly live (green) cells remained within the core of the biofilm structures (Figure 3A). Although biofilm bacteria tolerated the antibiotic treatments at the concentrations used (10 μM), free-living bacteria within the planktonic phase remained susceptible (Figure 3D).

Adjunctive NO used in combination with 5 μg ml^−1^ tobramycin (with or without ceftazidime) demonstrated a pronounced and significant reduction in *P. aeruginosa* mean biofilm biomass and thickness compared with both untreated biofilms and biofilms treated with antibiotics in the absence of NO (p = 0.001) (Figures 3B and 3C). Residual surface-attached biofilms observed by CSLM appeared as only a thin monolayer, indicating that the majority of the remaining surface-attached *P. aeruginosa* had been killed, as shown in Figure 3A by increased red fluorescent staining with propidium iodide. In addition, there was a marked reduction in viable planktonic cells following adjunctive NO treatments (Figure 3D), demonstrating that bacteria released from biofilms during NO-induced dispersal are killed in the planktonic phase by the combined antibiotic treatment.

### A Proof-of-Concept Randomized Trial Demonstrates that Low-Dose Nitric Oxide Adjunctive Therapy Reduces Detectable *P. aeruginosa* Biofilm in Patients without Increasing Planktonic Bacterial Loads

**Figure 4:**
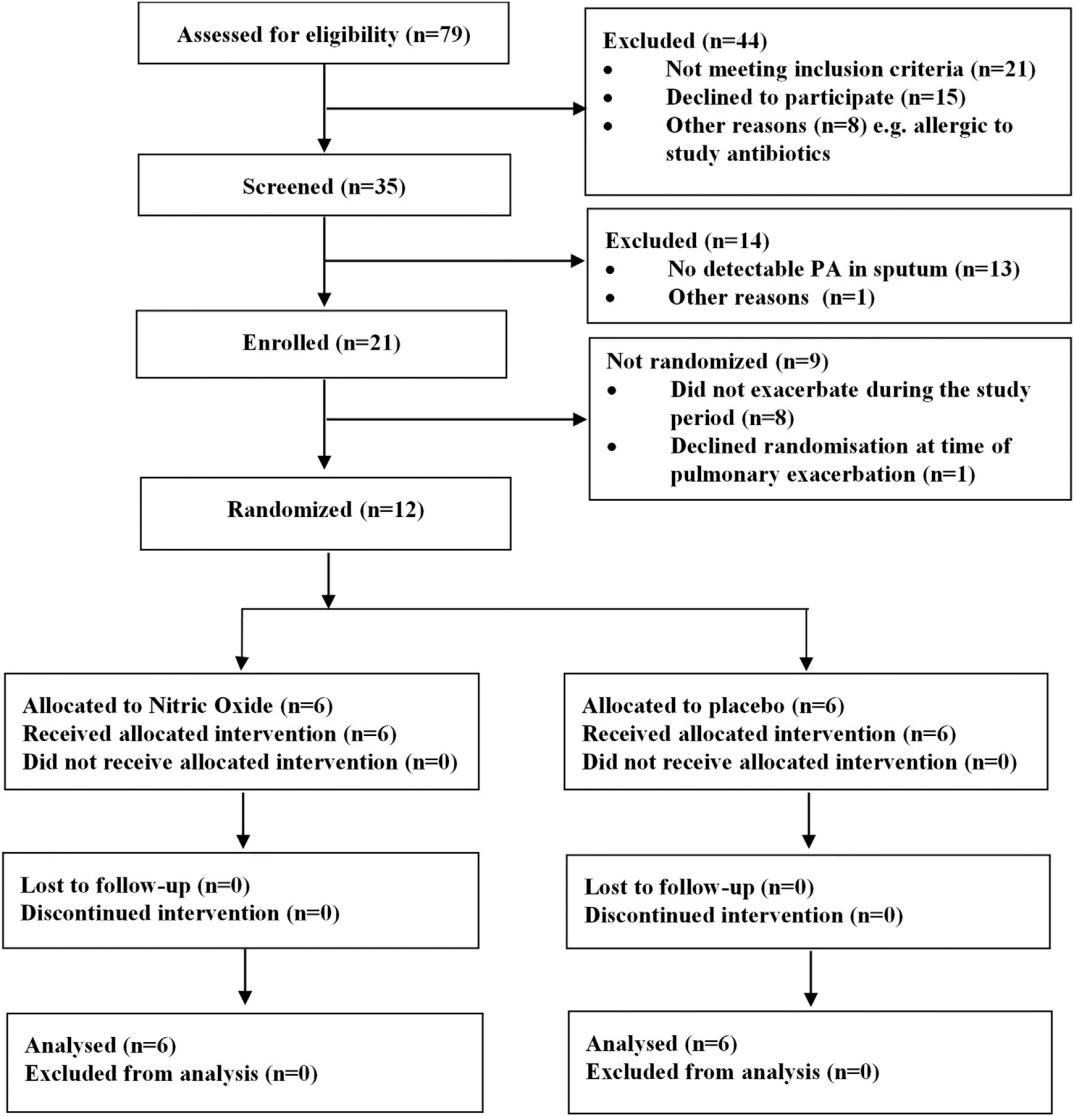
Clinical Study CONSORT Diagram Depicting the Flow of Patients through the Study For patients to be randomized, they had to be admitted during pulmonary exacerbation to receive trial therapy concurrently with i.v. antibiotics.

**Figure 5:**
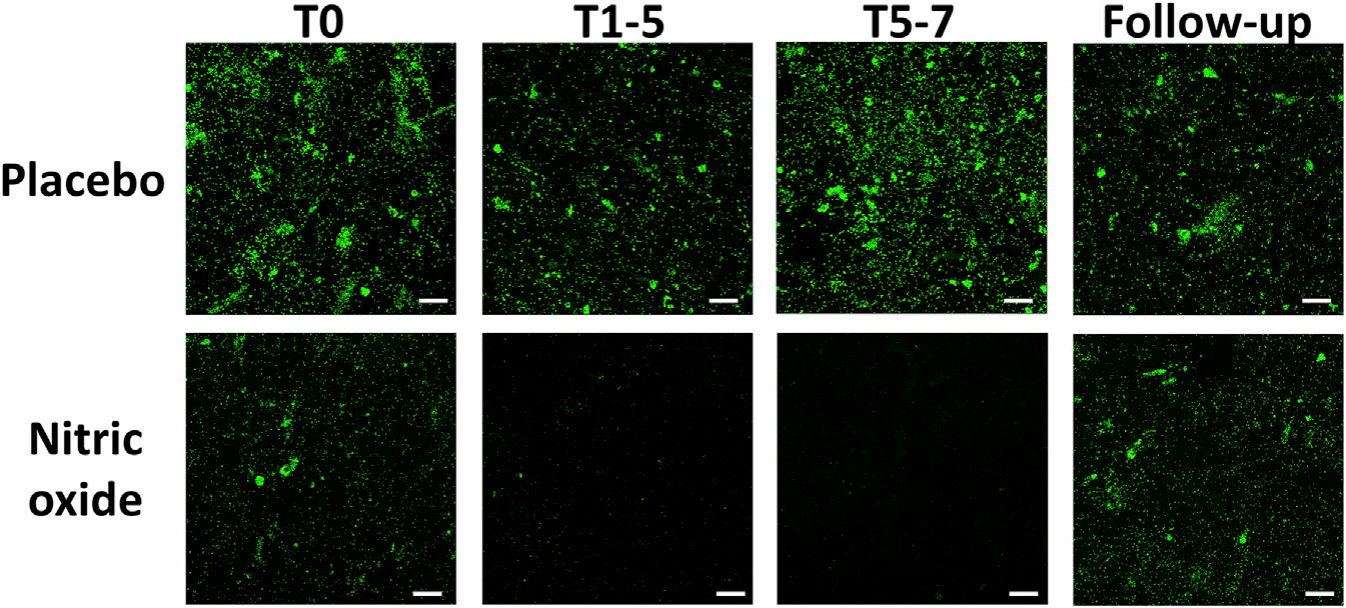
Reduction in *P. aeruginosa* Biofilm with NO Adjunctive Therapy Shown are representative FISH confocal images from a CF patient being treated with NO adjunctive to conventional antimicrobial agents (ceftazadime and tobramycin) compared with a patient on antibiotics alone (n = 6 in both the NO and placebo groups). Almost no *P. aeruginosa* biofilms were detectable in the treatment group compared with placebo. At follow-up, 10–13 days after NO adjunctive treatment stopped, pseudomonal biofilm was detected in sputum, having been reduced while on NO. Scale bars, 25 μm. The central panels show xy plan views of merged image stacks (total biofilm detected in 3D imaging), and the rectangular z axis side panels show representative single side views of the biofilm.^59^

12 patients were randomized to receive either low-dose NO inhalation or placebo (CONSORT diagram; Figure 4). Adjunctive NO used in combination with tobramycin and ceftazidime demonstrated a significant reduction in the key primary microbiological endpoint, *P. aeruginosa* biofilm aggregates. This is shown in aggregates both over 20 cells in size and in those over 10 cells in size compared with those receiving placebo with antibiotics over the 7 days of treatment (generalized estimating equations [GEE] analysis, p = 0.031 and p = 0.029, respectively, for days 5 and 7; Figure 5). The data suggested less *P. aeruginosa* biofilm, as quantified by both the number and volume of aggregates greater than 20 or 10 cells in the NO group compared with placebo through day 7 while on NO therapy. This reduction was not fully maintained after treatment was stopped; pseudomonal biofilm was detected in treatment group samples at a time point of 10–13 days following the cessation of NO therapy (study period days 5 through 20; Table 1 and Figure 5). See Materials and Methods for the rationale regarding cluster size selection. Other important secondary endpoints are shown in Table 2. From an individual participant safety perspective, there was no evidence that biofilm dispersal increased the amount of viable *P. aeruginosa* detected in planktonic phase by colony-forming units (CFUs). qPCR, indicative of total viable *P. aeruginosa* cells,^20^ did not demonstrate a difference between groups because of the small numbers and large variation between individuals. There were also no adverse clinical safety signals (FEV1, forced vital capacity [FVC], quality of life score) in the treatment group compared with those treated with placebo. Baseline clinical data are shown in Table 3, baseline laboratory data and study adverse effects are shown in Tables S1 and S2, and individual patient data for the primary outcome (FISH) and one clinical parameter (FEV1) are shown in Table S3.

**Table 1:** Primary Outcome Results Showing Mean Differences between the NO or Placebo Groups of Change from Baseline

	Change from Baseline, Mean (SD)				Treatment Effect: Mean Difference (95% CI), p Value			
Day	5	7	10	20	Intervention Period (Days 5 and 7)		Study Period (Days 5, 7, 10, and 20)	
**FISH: Ln Number of Aggregates > 20 Cells**								

Placebo	0.11 (2.38)	0.35 (1.44)	0.38 (2.32)	NA	3.49 (0.32, 6.67)	p = 0.031	1.35 (−0.58, 3.7)	p = 0.170
NO	−4.33 (5.11)	−2.19 (3.93)	0.98 (1.83)	NA				

**FISH: Ln Volume of Aggregates > 20 Cells**								

Placebo	−0.16 (2.51)	−0.03 (1.54)	0.21 (2.20)	NA	4.47 (−0.40,8.98)	p = 0.052	2.35 (0.08, 4.63)	p = 0.043
NO	−6.10 (7.50)	−3.03 (5.88)	0.97 (2.02)	NA				

**FISH: Ln Number of Aggregates > 10 Cells**								

Placebo	0.28 (2.09)	0.26 (1.52)	0.20 (2.04)	NA	2.44 (0.25, 4.62)	p = 0.029	1.09 (−0.54, 2.72)	p = 0.118
NO	−1.46 (1.08)	−2.71 (4.56)	1.10 (1.19)	NA				

**FISH: Ln Volume of Aggregates > 10 Cells**								

Placebo	0.08 (2.33)	0.05 (1.50)	−2.47 (2.12)	NA	2.68 (-.052, 5.41)	p = 0.055	1.27 (−0.62, 3.16)	p = 0.188
NO	−1.75 (1.14)	−3.37 (6.34)	1.07 (1.50)	NA				

CI, confidence interval; Ln, natural logarithm; NA, not analyzed.

**Table 2:** Microbiological and Clinical Safety Monitoring Showing Mean Differences between the NO or Placebo Groups of Change from Baseline

	Change from Baseline, Mean (SD)				Treatment Effect, Mean (95% CI), p Value			
Day	5	7	10	20	Intervention Period (Days 5 and 7)		Total Study Period (Days 5, 7, 10, and 20)	
**Ln CFU**								

Placebo	−1.62 (2.34)	−2 (3.77)	−0.89 (4.08)	NA	−0.19 (−2.95, 2.56)	p = 0.891	0.03 (−2.53, 2.59)	p = 0.980
NO	−1.97 (2.20)	−1.25 (2.76)	−1.30 (1.64)	NA				

**Ln qPCR**								

Placebo	−2.16 (1.73)	−4.33 (2.44)	−4.32 (1.92)	NA	−0.47 (−1.91, 0.97)	p = 0.519	−0.37 (−1.44, 0.71)	p = 0.504
NO	−1.86 (1.60)	−3.67 (1.81)	−3.09 (1.74)	NA				

Day	5	7	10	20	Intervention Period (Day 7 Only)		Study Period (Day 20 Only)	

**FEV1**								

Placebo	NA	6.67 (4.46)	9.00 (2.52)	6.17 (3.49)	−8.93 (−25.3, 7.42)	p = 0.248	1.95 (−7.31, 11.20)	p = 0.645
NO	NA	15.6 (17.2)	5.01 (14.2)	4.22 (9.35)				

**FVC**								

Placebo	NA	4.83 (6.74)	9.17 (5.46)	6.33 (4.46)	−11.6 (−30.7, 8.42)	p = 0.229	8.03 (−4.10, 20.2)	p = 0.168
NO	NA	16.0 (20.1)	3.75 (14.6)	−1.70 (12.3)

**Table 3:** Baseline Clinical Characteristics of Groups

	Treatment Group	n	Mean	SD
Age (years)	A	6	30.0	13.99
	B	6	29.3	15.60
Height (cm)	A	6	162.8	9.45
	B	6	166.0	9.27
Weight (kg)	A	6	56.4	9.61
	B	6	63.0	8.32
Heart rate (bpm)	A	6	89.3	18.62
	B	6	91.2	17.19
Systolic blood pressure (mmHg)	A	6	107.3	13.84
	B	6	121.0	14.97
Diastolic blood pressure (mmHg)	A	6	64.2	9.37
	B	6	75.8	13.73
Oxygen saturation (% in air)	A	6	95.2	2.23
	B	6	95.2	3.25
Respiratory rate (per minute)	A	6	20.0	1.10
	B	6	18.5	2.17
Temperature (°C)	A	5	36.8	.31
	B	6	36.9	.48
FEV1 % of predicted (l)	A	6	40.2	20.14
	B	6	45.7	18.28
FVC % of predicted (l)	A	6	54.4	17.60
	B	6	71.5	21.11
Average exhaled NO levels (ppb)	A	6	12.7	9.46
	B	6	9.3	8.86

A, NO group; B, placebo group.

### Circulating NO Metabolites Change Little during Low-Dose NO Inhalation in CF Patients

Plasma nitrate (NO_3_^−^) concentrations tended to increase in response to delivery of low-dose NO, but these changes did not reach statistical significance (p > 0.05). Plasma levels of nitrite (NO_2_^−^) and total nitrosation products (RXNO) paradoxically decreased during NO inhalation, although this was also not significant. With the exception of unusually high nitrite levels in erythrocytes compared with plasma values, there was also no obvious effect of inhaled NO on NO metabolite status in these blood cells, which is surprising given that nitrosylhemoglobin (NO-heme) is the most sensitive marker of NO availability in vivo, and nitrate is the final oxidation product of NO^21^ (thus, both might be expected to be elevated following prolonged NO inhalation). Direct NO measurement in sputum was impractical because of the short half-life of NO in relation to the time taken for the probe to equilibrate in individual sputum samples (data not shown). Overall, determination of a comprehensive panel of NO metabolites suggested that low-dose inhaled NO does not significantly affect circulating NO metabolites in CF (Figure S1).

## Discussion

Targeted therapy to address biofilm infection, rather than using conventional antibiotics alone, represents a potential paradigm shift in the treatment of chronic pseudomonal infection in cystic fibrosis. Our experiments show that adjunctive NO can disrupt *P. aeruginosa* biofilms and suggest a novel approach to the challenge of managing persistent *Pseudomonas* biofilm infection in CF patients.

The importance of the biofilm phenotype in promoting *P. aeruginosa* survival and persistence within the lower respiratory tract is well established.^4^, ^22^ However, there are currently no clinically recognized therapeutic approaches for eradicating established biofilm-associated *P. aeruginosa* respiratory infections. New treatment strategies for bacterial biofilms are a critical unmet need.^23^, ^24^, ^25^

Our approach was to design a clinical diagnostic platform that could be used to detect changes in *Pseudomonas* biofilm from patients with CF. We used FISH as a primary technique to identify biofilm in clinical samples, as recommended by the European Society of Clinical Microbiology and Infectious Diseases (ESCMID) guidelines for the diagnosis and treatment of biofilm infections.^25^ We first used ex vivo samples from CF patients to establish the diagnostic platform. We tested *P. aeruginosa* clinical isolates growing in biofilms and used FISH to follow the effects of NO on aggregate size in these biofilms. We designed the proof-of-concept clinical study to determine whether changes in the size of *Pseudomonas* biofilm aggregates taken from patients who had been given low-dose NO could be detected during and following treatment regimes.

Ex vivo studies demonstrated that low concentrations of NO (<500 nM) significantly reduced the amount of *P. aeruginosa* biofilm aggregates in CF sputum, potentiating the effect of the aminoglycoside antibiotic tobramycin alone and in combination with the third-generation cephalosporin ceftazidime.

In addition, our results suggest that adjunctive low-dose NO might prevent a previously reported potential biofilm-enhancing effect of aminoglycoside treatment.^13^ Our ex vivo data show that treatment of CF *P. aeruginosa* biofilms with clinically relevant concentrations of tobramycin can lead to increased biofilm growth. Bacteria in biofilms within the CF lung are likely to be exposed to sub-inhibitory concentrations of antibiotics because of poor penetration or diffusion gradients through the biofilm.^26^ Such sub-inhibitory antibiotic concentrations may explain the apparently paradoxical increase in biofilm thickness we observed despite increased cell death. It is possible that initially low antibiotic concentrations within the biofilm induce bacterial growth and/or extracellular matrix production, followed by increased cell death as the antimicrobial concentration increases because of diffusion into the biofilm. An alternative explanation for the increased biofilm thickness might be enhanced cell lysis, which has been shown to contribute to *P. aeruginosa* extracellular matrix production.^27^ Importantly, irrespective of mechanism, the observed enhancement of *P. aeruginosa* biofilm growth in the presence of tobramycin was completely eliminated in the presence of 450 nM adjunctive NO. Nitric oxide potentiated the effect of tobramycin alone, and ceftazidime and tobramycin in combination, by dispersing *P. aeruginosa* biofilms and facilitating the killing of dispersed bacteria.

The proof-of-concept clinical study demonstrated a significant direct effect on pseudomonal biofilm (as measured by a reduction in *P. aeruginosa* aggregate load) in CF patients treated with NO gas plus conventional intravenous (i.v.) antibiotic therapy compared with i.v. antibiotics alone. The effect was not sustained following the end of NO therapy in this group of adult patients with long-term chronic disease. In this small study, we did not detect any side effects as a result of this treatment strategy. All changes in the clinical parameters measured favored NO treatment, and there was no evidence that NO treatment caused an increase in overall bacterial load or the severity of acute exacerbations. We saw no treatment effects suggestive of NO induced vasodilatation (i.e., no increase in oxygen saturation during treatment) and no adverse effects during the weaning period at the end of each day’s NO therapy that might have been indicative of rebound pulmonary hypertension. Our study measured clinical parameters to ensure safety but not clinical efficacy, which will be the subject of future large clinical trials.

Previous studies have shown that *P. aeruginosa* cells can be killed directly by high doses of NO.^28^ This might be the result of several possible toxic effects of NO on bacteria at high concentrations, including direct modification of membrane proteins, DNA cleavage, and lipid peroxidation through mechanisms of both nitrosative and oxidative stress.^29^, ^30^, ^31^ The use of high-dose NO in this way has potential cytotoxic and other adverse clinical effects^32^ and is associated with considerable cost. Despite this, recent trials of high-dose 160-ppm inhaled NO in CF did not demonstrate any adverse safety signals.^33^, ^34^ In terms of biofilm growth, which has not been measured in previous clinical trials, high levels of NO might result in increased nitrate levels in CF sputum that may support growth of *P. aeruginosa* by metabolism based on anaerobic denitrification.^35^, ^36^ Our previous in vitro studies have shown that higher concentrations of NO can stimulate biofilm formation.^18^ These studies agree with another report suggesting that higher-dose NO may, in fact, enhance aminoglycoside tolerance by blocking energy-dependent phases of drug uptake.^37^ The low-dose, signal-relevant concentrations of NO we used in the proof-of-concept clinical trial reported here are approximately three orders of magnitude lower than those shown to inhibit drug uptake and did not inhibit tobramycin efficacy against dispersed (planktonic) or biofilm *P. aeruginosa* bacteria.

The rationale for our approach using NO to treat *P. aeruginosa* infection was to exploit our discovery that low-dose NO (10 ppm, assumed to translate into submicromolar concentrations locally) mediates biofilm dispersal through increased bacterial phosphodiesterase activity and an associated decrease in c-di-GMP levels.^19^ We have previously shown that low-dose NO can increase the motility of *P. aeruginosa* cells in vitro^18^ and proposed that this increased motility promotes biofilm dispersal. In contrast, and in the context of CF sputum, other studies have shown that *P. aeruginosa* isolates are frequently non-motile^38^ and that sputum can repress *P. aeruginosa* flagellar activity and motility.^39^, ^40^ Cyclic-di-GMP binds to a broad range of effector components that control the physiology, development, stability, cell adhesiveness, and motility of the biofilm phenotype. Factors other than motility could therefore be responsible for biofilm disruption and a reduction in tolerance to antibiotic treatment. Further studies are required to understand the specific c-di-GMP effectors responsible for NO-mediated disruption of biofilms within CF sputum.

Chronic CF infections are often associated with multiple bacterial pathogens and complex microbial communities.^41^, ^42^ Genes that modulate c-di-GMP turnover are widely distributed in bacteria, and NO-mediated dispersal has now been observed across a number of species, including many pathogenic organisms.^43^, ^44^ NO-mediated alteration of intracellular c-di-GMP levels is therefore an important new potential target to control multispecies bacterial communities in CF. NO might also be of benefit in treating younger CF patients after initial infection with *P. aeruginosa*. Used under these circumstances, it might increase the effectiveness of eradication therapy and delay the onset of chronic biofilm infection with this organism.

Our clinical trial data appear to differ from the reported effects of inhaled NO on circulating NO metabolite levels in infants with pulmonary hypertension,^45^ where a clear increase in NO metabolite levels was reported to occur with twice the concentration of inhaled NO used in our study. There is a paucity of information on circulating levels of NO metabolites in CF. Nevertheless, our observations are in general agreement with the notion that NO concentrations are lower in the exhaled breath of CF patients, whereas systemic NO production does not appear to be compromised.^46^ Possible mechanisms for this include accelerated degradation as a result of increased oxidative stress in epithelial cells, increased NO consumption by bacterial biofilms, or impaired gas exchange as a result of mucus obstruction. All of these factors would be expected to prevent exogenous inhaled NO to reach the systemic circulation, limiting its effects to the site of administration.

The main limitation of the clinical component of our study is the small sample number and between-patient variation in clinical and microbiological parameters. This has made formal statistical analyses difficult, but we were able to incorporate repeated measurements over time to improve power. Variability in the qPCR results between the NO and placebo groups was probably due to sample heterogeneity in chronically infected patients. Despite these limitations, FISH image analysis data demonstrate a treatment effect and provide a proof of concept for our low-dose NO approach. Similarly, our analysis of the changes in systemic NO status following low-dose NO inhalation is likely compounded by inter-individual differences in NO processing. However, the lack of an observed rise in blood nitrate and NO-heme levels are consistent with well-documented perturbations in NO production and handling in CF patients.^47^, ^48^

Our study has demonstrated the potential for the use of low-dose NO to enhance antibiotic treatment of biofilm infections. Although the practical challenges in delivering inhaled NO gas to CF patients were considerable, future novel NO donor antibiotics might prove to be a more feasible approach to targeting biofilms.^49^ Biofilm-related chronic infections are responsible for at least half a million deaths per year at an estimated cost of over $94 billion in the United States alone.^16^ More effective anti-biofilm therapies are needed to address this significant unmet need.

## Materials and Methods

### CF Sputum Collection and *P. aeruginosa* Isolation

Sputum samples^50^, ^51^ from 72 patients with CF (median age at informed consent, 21 years; range, 17–62; United Kingdom National Health Service [NHS] Research Ethics Reference 08/H0502/126) were obtained by CF physiotherapist-assisted sample expectoration. For isolation of *P. aeruginosa* from sputa, samples were digested using Mucolyse (Pro-Lab Diagnostics) containing dithiothreitol and phosphate buffer for 15 min at 37°C, followed by culture on *P. aeruginosa*-specific cetrimide agar (Sigma-Aldrich). Multiplex PCR was used to confirm *P. aeruginosa* as described previously.^52^ Because *P. aeruginosa* colonization of the CF lung often consists of multiple clonal lineages,^53^ colony sweeps (sterile loops drawn across a confluent streak of bacterial growth on cetrimide agar) were used in preference to single-colony isolates for routine subculture and biofilm growth of *P. aeruginosa*.

### Nitric Oxide-Mediated Dispersal of Clinical *P. aeruginosa* Isolates

We first evaluated the ability of NO at different doses to disperse clinical isolates of *P. aeruginosa* biofilms in vitro and within sputum from CF patients. Biofilm-forming *P. aeruginosa* clinical isolates (n = 12) were inoculated using overnight cultures grown in M9 minimal medium (20 mL per liter of 20% glucose, 2 mL per liter of 1 M MgSO_4_, and 100 μL per liter of 1 M CaCl_2_). Overnight cultures were diluted to give OD readings corresponding to 10^6^ cells/mL, and 200 μL aliquots were inoculated into a 96-well plate and incubated at 37°C for 24 hr. The medium was aspirated and replaced with fresh M9 medium with or without increasing concentrations of the NO donor SNP (concentration range, 9 pM to 4.5 μM; Sigma Aldrich). The concentration of NO produced by SNP was calculated using an NO microsensor (Unisense) and calibrated over a range of 250 nM to 10 μM using previously published methods.^54^ Based on the measured linear relationship between micromolar concentration of SNP producing nanomolar concentrations of NO (where y = 0.9022x; R^2^ = 0.9617, n = 6 data points), NO concentrations were calculated to be nearly 1,000-fold less than the starting concentration of SNP, resulting in approximately 450 nM NO generated from 500 μM SNP. To confirm that the effects were specific to NO, the assays were also carried out with SNP (500 μM) in the presence of 5 mM of the NO scavenger carboxy-PTIO (Sigma-Aldrich). M9 medium containing 500 μM potassium ferricyanide (Sigma-Aldrich), used to generate breakdown products of SNP,^55^, ^56^ was also used as a control. Optical density measurements of the supernatant containing planktonic cells were made using a BMG Labtech Omega plate reader (620 nm and chamber temperature of 37°C) over 24 hr, with measurements taken every 15 min. Experiments were repeated three times with four replicates for each experiment.

### Nitric Oxide-Mediated Dispersal of *P. aeruginosa* Biofilms in CF Sputum and Antibiotic Sensitivity Testing

The use of FISH to identify microbial biofilms in situ is recommended in the ESCMID guidelines for the diagnosis and treatment of biofilm infections.^25^ Expectorated sputum samples (n = 5) were divided in half (v/v) and treated for 15 hr with either Hank’s balanced salt solution (HBSS, Sigma-Aldrich) alone or HBSS containing 450 nM NO (i.e., 500 μM SNP). Samples were fixed in freshly prepared 4% paraformaldehyde in PBS at 4°C and washed with PBS and PBS-ethanol (1:1 v/v), and 20-μL drops of sputum were spotted onto poly-L-lysine (PLL)-coated slides and left to dry overnight. *P. aeruginosa* detection was performed using FISH with the following 16S ribosomal probe sequences: PseaerA, 5′-GGTAACCGTCCCCCTTGC-3′, specific for *P. aeruginosa*,^57^ labeled with Cy3; EUB338, 5′- GCTGCCTCCCGTAG GAGT-3′ (domain bacteria),^58^ labeled with Cy5 (Integrated DNA Technologies). Hybridization conditions for FISH were optimized and stringently evaluated in vitro to ensure the specificity of the PseaerA probe. We independently confirmed the previously reported optimal hybridization conditions for the specificity of the Pseaer probe for *P. aeruginosa*.^57^, ^59^ Hybridization with the sample was carried out using 20% formamide, and a 2-hr incubation at 46°C was followed by washing for 15 min at 48°C in pre-warmed wash buffer as described previously.^57^, ^60^ Coverslips were placed on samples and imaged using an inverted DMI600 SP5 confocal laser-scanning microscope (CLSM, Leica Microsystems). Control experiments with both positive and negative controls demonstrated that low concentrations of NO in the concentration range used for our studies did not interfere with the eubacterial or species-specific FISH signal for *P. aeruginosa*, including no fluorescence quenching in the presence of NO (Figure S2).

*P. aeruginosa* biofilms were examined for antibiotic sensitivity using adjunctive treatment of 450 nM NO with or without the aminoglycoside tobramycin. The antibiotic was added alone or in combination with the cephalosporin ceftazidime (both antibiotics at the minimum bactericidal concentrations [MBCs] to induce killing of planktonic cells, determined to be 5 μg ml^−1^). Biofilms were grown from colony sweeps as described above in culture plates (MatTek), and treatment was carried out for 15 hr at 37°C. Ceftazidime is not used alone to treat CF exacerbations because of the emergence of resistant bacterial strains and so was used only in combination with tobramycin in this study. Viable bacterial cell counts were determined on cetrimide agar, and residual surface bound biofilms were examined using a CLSM and the Baclight Live/Dead viability stain (Invitrogen).

### Proof-of-Concept Randomized Clinical Trial

We subsequently conducted a randomized, participant- and outcome assessor-blind, placebo-controlled proof-of-concept study of inhaled NO gas in hospitalized participants aged 12 and above with CF and chronic *Pseudomonas* colonization between August 2011 and September 2012 (UK NHS REC 11/H0502/7, EudraCT 2010-023529-39, ClinicalTrials.gov NCT02295566) (CONSORT diagram; Figure 4).

### Study Design and Placebo

The design for the proof of concept was randomized and placebo-controlled, where participants and primary outcome assessors were blind to the treatment group. Participants randomized to the placebo arm of the trial received medical air (BOC) or a medical air/oxygen blend according to clinical need (determined by oxygen saturation monitoring according to standard clinical practice). This was administered through a nasal cannula in the same manner as the NO so that participants did not know whether they received the trial treatment or placebo, including pre-defined sham weaning procedures.

### Sample Size and End of Study

The primary aim of this study was to gain evidence that NO could reduce the proportion of aggregated bacteria in biofilms (with regard to reduction in surface area and reduction in average colony size) in the sputum of participants treated with NO. To demonstrate that treatment with NO is better than the control, we calculated the sample size required to achieve a 90% probability of observing the correct ordering (consistent with a treatment effect) of the proportion of bacteria in biofilms for each group (estimated by taking into account the results observed from the laboratory experiments).^61^ It was estimated that the proportion of bacteria in biofilms with regard to surface area (as a measure of aggregate size) would be 0.7 for placebo and 0.4 for patientrs treated with NO. A sample size of ten participants in each treatment group would have been sufficient to determine that the NO treatment arm is superior to the control group (by reducing the proportion of biofilm bacteria) with 90% probability, assuming a change from 0.7 to 0.4. It was recognized that this study would have a limited ability to detect important but rare treatment-related adverse events that would need to be identified in a future larger randomized control trial (RCT). The study was ended at the end of the funding period, when six participants had been recruited to each group. The data were analyzed according to the statistical plan despite the lower-than-expected recruitment.

### Inclusion and Exclusion Criteria

Adolescents and young adults with cystic fibrosis were eligible for inclusion when aged 12 or above and colonized with *P. aeruginosa*, confirmed by microbiological assessment of sputum samples. Patients were excluded for colonization with *Burkholderia cepacia*; known hypersensitivity to the antibiotics used in the study; other known contraindications to the antibiotics to be used in the study, including known aminoglycoside-related hearing/renal damage; patients requiring non-invasive ventilation; patients who had a pneumothorax; patients who were admitted for specific treatment of nontuberculous mycobacteria; patients who could not tolerate a nasal cannula (e.g., those who could not breathe through the nose); patients who had nasal polyposis, causing significant blockage of the nasal passages; adolescents not Gillick-competent (and therefore not able to give their own assent in addition to parental consent); patients not likely to survive the time period of the study washout period (4 months from enrolment); treatment with an investigational drug or device within the last 3 months prior to enrolment; patients who were pregnant (a pregnancy test was carried out for females 11 years of age and older); and immediate families of investigators or site personnel directly affiliated with the study. Immediate family was defined as child or sibling, whether biological or legally adopted.

### Study Intervention and Randomization

Nitric oxide gas (10 ppm; INOmax, 400 ppm mol/mol inhalation gas; INO Therapeutics), delivered via INOvent (Ikaria, supplied by INO Therapeutics), or identically delivered placebo (air or air/oxygen mix) was administered via nasal cannula to 12 participants admitted for i.v. antibiotics to treat pulmonary exacerbations. The study intervention was administered by inhalation via nasal cannula for 8 hr overnight for the first 5–7 days of i.v. antibiotic therapy. This dose was based on extrapolation from in vitro work, also informed by the low dose used in hypoxic respiratory failure associated with evidence of pulmonary hypertension in preterm infants. Electrochemical measurement of NO gas released in solution by approximately 500 μM SNP was measured to be around 390 nM NO,^19^ which is equivalent to 390 nmol/L, giving 8.7 μL/L or 8.7 ppm (not taking into account any adjustment because of the environmental temperature). Participants and medical and laboratory staff were blinded to treatment allocation. Block randomization with block lengths 2 and 4 was undertaken via an online randomization service in a 1:1 ratio to ensure concealment of treatment allocation. Participants were monitored closely by a research nurse during the overnight study intervention period, and monitoring and safety data were collected.

### Clinical Study Outcomes

The primary outcome was the between-group difference in proportion of bacteria in biofilms (as determined by direct visualization of the biofilm by FISH^57^, ^58^, ^59^, ^60^ and image analysis). Secondary outcomes were between-group differences in CFUs and qPCR^20^; measures to assess safety, including lung function (FEV1 and FVC); and health-related quality of life assessment (CFQ-UK)^62^.

### Determination of Nitric Oxide in Sputum

We attempted to determine the free NO concentrations in expectorated sputum samples following inhaled NO therapy by using a NO electrochemical probe (Unisense NO microsensor, glass sensor NO-10). However, because of difficulties in equilibrating and calibrating the probe within CF sputum and insufficient volumes of sputum produced by patients to carry out NO measurements alongside FISH and molecular analyses, these data are not presented.

### Image Analysis

For the ex vivo experiments, quantification of *P. aeruginosa* biofilm thickness and biomass was made from 3D CSLM stacks using the freely available COMSTAT^63^ software. To avoid subjectivity in the selection of sample regions, treatment groups were blinded to the researchers carrying out the sample analysis. To specifically avoid subjective bias, sample areas selected for study were chosen in a predetermined pattern. Means and SD were calculated from five random fields of view per treatment group. For clinical trial samples, FIJI (http://fiji.sc/) 3D object counter software was used to analyze and quantify *P. aeruginosa* “biovolume” analysis of confocal stacks. The range of volumes of a single *P. aeruginosa* cell from the literature^64^ (0.16–3.67 μm^3^) was used to filter fluorescently labeled objects in the stacks into the following groups: noise (all objects below single-cell size, estimated as less than 0.16 μm^3^); single cells; clusters (aggregates) over 10 cells in volume; and clusters over 20 cells in volume. After thresholding, the volume of a *P. aeruginosa* cell was assessed using the 3D object counter and compared with literature values for concordance. The 3D object counter was then used to record all objects in each sample, and the results for each of the ten image stacks per sample were collated into databases and grouped for analysis. For the primary analysis, aggregated cell clusters containing both more than 20 cells and more than 10 cells in size were selected because all patients had microcolonies over this size at baseline, so changes could be seen over the time course of the study. There were not enough clusters of more than 40 cells to analyze; however, because the 20-cell-sized microcolonies were estimated using the upper limit of a *Pseudomonas aeruginosa* (PA) cell size based on literature values (3.67 μm^3^), aggregates of more than 20 cells by our definition were likely to contain more than 20 cells.

### Measurement of Nitric Oxide Metabolites in the Blood

Venous blood was collected in EDTA tubes 1 and 7 hr after starting inhaled NO/placebo therapy on day 1 and immediately separated into plasma and blood cells by centrifugation for 10 min at 800 × *g*; aliquots of plasma and red blood cell (RBC) pellets were snap-frozen in liquid nitrogen and stored at −80°C until analysis. NO metabolite concentrations in the plasma and RBC lysate were quantified immediately after thawing of frozen samples in the presence of excess N-ethylmaleimide (in PBS, 10 mM final concentration) as described previously.^65^, ^66^, ^67^ Briefly, nitrite and nitrate were quantified simultaneously via high-pressure liquid ion chromatography (ENO-20, Eicom) with post-column Griess diazotization following on-line reduction of nitrate to nitrite. Total nitrosation products (including low-molecular-weight S-nitrosothiols, N-nitrosamines, and nitrosated proteins) were measured using group-specific de-nitrosation/reduction and subsequent liberation of NO, detected using gas phase chemiluminescence (CLD77am sp, Ecophysics). NO-heme concentrations were quantified by injection of RBC lysate into an oxidizing reaction solution (ferricyanide in PBS),^67^ and generated NO was quantified by gas phase chemiluminescence as above.

### Statistical Analysis

Data for the laboratory study were compared using a Mann-Whitney rank sum test for non-normally distributed data. For the clinical study, an intention-to-treat analysis was undertaken.

For all outcomes, the change from baseline to endpoint was calculated. The primary outcome (FISH, the number and volume of aggregates >20 cells) and microbiological and clinical safety outcomes (CFUs and qPCR) were analyzed on the natural log scale.

The mean difference of the treatment effect between arms during the intervention period (days 5 and 7) and total study period (days 5, 7, 10, and 20) was estimated by conducting linear regression using the method of GEE^68^ to account for longitudinal dependence (where study time points were available). Residuals were examined to assess model assumptions. Analyses were performed in Stata software, version 11.

## Author Contributions

The project was conceived by S.C.C., S.N.F., and J.S.W. K.C. wrote the protocol first draft and led regulatory applications. R.P.H., L.H.S., and P.S. led laboratory method development. R.P.H. carried out microbiological data acquisition. For the clinical study, S.N.F. acted as chief investigator and G.C. and T.D. as pediatric and adult clinical principal investigators, respectively. V.C. was the study statistician and J.S.W. the laboratory lead investigator. Additional biofilm and microbiology laboratory expertise and analysis were provided by C.D., R.N.A., N.B., K.D.B., J.J., M.K., S.K., S.A.R., G.B.R., and S.C.C. Clinical trial staff and investigators included S.P., C.S., P.S.S., R.S., J.L., M.C., and T.D. Nitric oxide metabolite assays and expertise were provided by M.F. and B.O.F.

## Supplemental Information

Document S1. Figures S1 and S2 and Tables S1–S3

Document S2. Article plus Supplemental Information
