# Supplementary material for: Low-Dose Nitric Oxide as Targeted Anti-biofilm Adjunctive Therapy to Treat Chronic Pseudomonas aeruginosa Infection in Cystic Fibrosis
Source: Mol Ther. 2017 Jul 24;25(9):2104–16. doi: 10.1016/j.ymthe.2017.06.021 (PMC5589160; doi:10.1016/j.ymthe.2017.06.021)

YMTHE, Volume *25*

Supplemental Information

Low-Dose Nitric Oxide as Targeted Anti-biofilm Adjunctive Therapy to Treat Chronic *Pseudomonas aeruginosa* Infection in Cystic Fibrosis

Robert P. Howlin, Katrina Cathie, Luanne Hall-Stoodley, Victoria Cornelius, Caroline Duignan, Raymond N. Allan, Bernadette O. Fernandez, Nicolas Barraud, Ken D. Bruce, Johanna Jefferies, Michael Kelso, Staffan Kjelleberg, Scott A. Rice, Geraint B. Rogers, Sandra Pink, Caroline Smith, Priya S. Sukhtankar, Rami Salib, Julian Legg, Mary Carroll, Thomas Daniels, Martin Feelisch, Paul Stoodley, Stuart C. Clarke, Gary Connett, Saul N. Faust, and Jeremy S. Webb

Supplementary Materials

**Table S1**	Baseline laboratory characteristics of groups (A=Nitric Oxide, B=Placebo)
**Table S2**	Clinical study adverse events and adverse reactions.
**Table S3**	Individual patient data before and after treatment with NO
**Fig. S1**	NO metabolite concentrations in blood plasma and erythrocytes for CF patients
**Fig S2**	NO does not affect FISH identification of *P. aeruginosa*

Table S1. Baseline laboratory characteristics of groups (A=Nitric Oxide, B=Placebo)

	Treatment Group	N	Median	IQR
**Hemoglobin**	A	6	132	118.5 - 138.8
	B	6	141	129.5 - 151.8
**White cell count**	A	6	7.9	6.6 - 11.7
	B	6	9.4	7.3 - 11.9
**Neutrophils**	A	6	6.2	3.6 - 8.8
	B	6	6.3	4.8 - 8.5
**Lymphocytes**	A	6	1.9	1.1 - 2.2
	B	6	2.0	1.4 - 2.4
**Platelets**	A	6	259	202 - 277
	B	6	248	228 - 297
**Sodium**	A	5	138	134.5 - 139
	B	6	138.5	136 - 139.2
**Potassium**	A	5	4.2	3.6 - 4.5
	B	6	3.6	3.3 - 4.3
**Urea**	A	5	5.2	4 - 6.1
	B	6	3.7	3.0 - 4.4
**Creatinine**	A	5	59	43.5 - 73.5
	B	6	57.5	46 - 72.5
**Total protein**	A	5	76	66.5 - 76.5
	B	6	73.5	69.3 - 81.5
**Albumin**	A	5	36	34 - 37.5
	B	6	37	34 - 40.3
**Bilirubin**	A	5	9	7 - 11.5
	B	6	9.5	7.5 - 11.8
**ALT**	A	5	20	19 - 28
	B	6	22.5	17.8 - 31
**ALP**	A	5	86	76.5 - 194.5
	B	6	83.5	68 - 128
**CRP**	A	5	37	13.5 - 41.5
	B	6	21	12 - 49
**Calcium**	A	5	2.4	2.3 - 2.4
	B	5	2.3	2.2 - 2.4
**Corrected calcium**	A	5	2.4	2.3 - 2.4
	B	5	2.3	2.3 - 2.4

Table S2. Clinical study adverse events and adverse reactions.

Subject number	Treatment group	Description of event	Occurred during therapy?	AE/AR	SAE	Intensity	Expectedness	Causality assessment
**R01**	Placebo	Epistaxis	Follow-up	AE	N	Mild	Not expected	Unlikely to be related
**R02**	NO	Cough and cold	Follow-up	AE	N	Mild	Not expected	Unlikely to be related
**R03**	NO	Increased cough, reduced appetite	Follow-up	AR	N	Mild	Not expected	Possibly related
**R03**	NO	Reduced O2 levels during therapy	Therapy	AE	N	Mild	Not expected	Unlikely to be related
**R01**	Placebo	Epistaxis	Follow-up	AE	N	Mild	Not expected	Unlikely to be related
**R05**	NO	Epistaxis	Therapy	AR	N	Mild	Not expected	Possibly related
**R05**	NO	Haemoptysis	Therapy	AR	N	Mild	Not expected	Possibly related
**R06**	NO	Tobramycin (clinical error, extra dose given at t=12 on day 1 and then 24 hourly thereafter)	Therapy	AE	N	Mild	Not expected	Not related

Table S3. Individual patient data before and after treatment with NO

S3a Summary tables for Ln FISH data – over 20 cell and over 10 cell size cluster data (per cm^3), data for absolute number of objects and biovolume shown.

Ln number of objects over 20 cell size

day	T0	T5	T7	T10	T20
**NO**	11.68	0.00	11.15	13.11	12.24
	10.16	10.77	10.50	11.97	
	10.75	9.11	8.99	11.94	10.84
	11.54	10.63	12.40	12.97	12.22
	12.14	9.60	9.87	9.53	13.05
	9.84	0.00	0.00	12.43	
**mean**	11.017	10.027	10.581	11.992	12.09
**95%CI**	10.058	9.184	9.227	10.624	10.631
	11.976	10.871	11.935	13.359	13.547
**SD**	0.914	0.804	1.290	1.303	0.916
**Placebo**	8.93	9.75	10.98		
	14.00	13.12	14.13	12.20	13.37
	10.61	9.01	10.04	9.90	10.76
	9.97	14.49	11.26	14.22	
	11.98	11.74	13.11	12.53	11.82
	11.56	9.58	9.65	11.17	10.51
**mean**	11.176	11.281	11.528	12.004	11.61
**95%CI**	9.322	8.968	9.689	10.005	9.543
	13.030	13.593	13.368	14.003	13.686
**SD**	1.767	2.204	1.753	1.610	1.302

Change in Ln number of objects over 20 cell size

day	T0	T5	T7	T10	T20
**NO**	11.68	-11.68	-0.53	1.43	0.57
	10.16	0.61	0.34	1.81	
	10.75	-1.64	-1.75	1.20	0.10
	11.54	-0.92	0.85	1.43	0.68
	12.14	-2.54	-2.27	-2.61	0.91
	9.84	-10.51	-10.51	2.59	
**mean**	11.017	-4.447	-2.312	0.975	0.565
**95%CI**	10.058	-9.972	-6.708	-0.938	0.023
	11.976	1.079	2.084	2.888	1.107
**SD**	0.914	5.265	4.189	1.823	0.341
**Placebo**	8.93	0.82	2.05		
	14.00	-0.88	0.12	-1.81	-0.63
	10.61	-1.60	-0.56	-0.71	0.16
	9.97	4.52	1.29	4.25	
	11.98	-0.24	1.13	0.55	-0.16
	11.56	-1.98	-1.92	-0.39	-1.06
**mean**	11.176	0.107	0.352	0.378	-0.423
**95%CI**	9.322	-2.392	-1.163	-2.506	-1.273
	13.030	2.605	1.866	3.262	0.428
**SD**	1.767	2.381	1.443	2.323	0.535

Ln total biovolume of objects over 20 cell size

day	T0	T5	T7	T10	T20
**NO**	16.55	0.00	16.34	18.29	17.40
	15.79	15.65	15.36	17.01	
	15.30	13.85	14.08	16.70	15.65
	16.25	15.46	17.25	17.67	17.18
	17.37	14.51	14.86	14.44	18.22
	14.80	0.00	0.00	17.79	
**mean**	16.012	14.870	15.578	16.985	17.111
**95%CI**	15.047	13.987	14.270	15.547	15.406
	16.976	15.752	16.886	18.422	18.815
**SD**	0.919	0.841	1.246	1.370	1.071
**Placebo**	14.05	14.56	15.48		
	19.25	18.27	19.17	17.42	18.35
	15.29	13.41	14.39	14.35	15.45
	15.38	19.87	16.37	19.30	
	17.18	16.63	18.22	17.35	16.96
	16.71	14.17	14.07	16.43	15.66
**mean**	16.310	16.152	16.284	16.968	16.604
**95%CI**	14.401	13.475	14.122	14.738	14.474
	18.219	18.830	18.445	19.198	18.734
**SD**	1.819	2.551	2.060	1.796	1.339

Change in Ln total biovolume of objects over 20 cell size

day	T0	T5	T7	T10	T20
**NO**	16.55	-16.55	-0.21	1.75	0.85
	15.79	-0.14	-0.43	1.22	
	15.30	-1.45	-1.23	1.40	0.35
	16.25	-0.79	1.00	1.41	0.92
	17.37	-2.86	-2.52	-2.93	0.84
	14.80	-15.66	-15.66	2.99	
**mean**	16.012	-6.242	-3.175	0.973	0.740
**95%CI**	15.047	-14.320	-9.709	-1.143	0.322
	16.976	1.837	3.359	3.090	1.158
**SD**	0.919	7.698	6.226	2.017	0.262
**Placebo**	14.05	0.51	1.42		
	19.25	-0.98	-0.08	-1.83	-0.91
	15.29	-1.88	-0.90	-0.94	0.16
	15.38	4.49	0.99	3.91	
	17.18	-0.55	1.04	0.17	-0.22
	16.71	-2.54	-2.64	-0.28	-1.04
**mean**	16.310	-0.158	-0.028	0.206	-0.503
**95%CI**	14.401	-2.793	-1.645	-2.530	-1.409
	18.219	2.477	1.588	2.942	0.404
**SD**	1.819	2.511	1.540	2.203	0.570

Ln number of objects over 10 cell size

day	T0	T5	T7	T10	T20
NO	12.58	10.20	11.47	14.06	12.76
	10.95	11.33	11.27	12.89	
	11.95	9.80	8.99	12.51	11.98
	12.26	11.57	13.48	13.79	12.83
	12.83	10.86	10.56	11.78	13.74
	10.75	8.84	0.00	12.88	
**mean**	11.887	10.432	9.295	12.984	12.83
**95%CI**	10.986	9.353	4.279	12.106	11.681
	12.788	11.511	14.312	13.861	13.975
**SD**	0.859	1.028	4.780	0.836	0.721
Placebo	9.62	10.85	12.08		
	14.52	13.87	14.89	12.82	14.13
	12.07	10.96	10.33	11.28	11.57
	11.07	15.13	11.96	14.68	
	12.82	12.59	13.57	13.12	12.47
	12.10	10.50	10.90	11.69	11.46
**mean**	12.035	12.315	12.289	12.718	12.41
**95%CI**	10.306	10.340	10.512	11.058	10.447
	13.763	14.289	14.066	14.377	14.371
**SD**	1.647	1.882	1.693	1.336	1.233

Change in Ln number of objects over 10 cell size

day	T0	T5	T7	T10	T20
NO	12.58	-2.38	-1.11	1.48	0.18
	10.95	0.39	0.33	1.94	
	11.95	-2.15	-2.96	0.56	0.03
	12.26	-0.69	1.22	1.53	0.57
	12.83	-1.98	-2.27	-1.06	0.91
	10.75	-1.92	-11.46	2.12	
**mean**	11.887	-1.455	-2.708	1.095	0.423
**95%CI**	10.986	-2.587	-7.496	-0.150	-0.209
	12.788	-0.323	2.079	2.340	1.054
**SD**	0.859	1.079	4.562	1.186	0.397
Placebo	9.62	1.22	2.46		
	14.52	-0.66	0.37	-1.70	-0.39
	12.07	-1.12	-1.74	-0.79	-0.50
	11.07	4.06	0.89	3.61	
	12.82	-0.23	0.75	0.30	-0.35
	12.10	-1.60	-1.20	-0.41	-0.64
**mean**	12.035	0.278	0.255	0.202	-0.470
**95%CI**	10.306	-1.913	-1.345	-2.327	-0.677
	13.763	2.470	1.855	2.731	-0.263
**SD**	1.647	2.089	1.525	2.037	0.130

Ln total biovolume of objects over 10 cell size

day	T0	T5	T7	T10	T20
**NO**	17.01	13.87	16.43	18.66	17.58
	15.99	15.89	15.73	17.41	
	16.08	14.14	14.08	17.00	16.30
	16.65	15.95	17.82	18.13	17.43
	17.63	15.13	15.15	15.88	18.47
	15.22	13.09	0.00	17.92	
**mean**	16.431	14.681	13.201	17.500	17.449
**95%CI**	15.541	13.462	6.288	16.470	16.033
	17.322	15.900	20.115	18.531	18.864
**SD**	0.848	1.162	6.588	0.982	0.889
**Placebo**	14.26	15.20	16.17		
	19.42	18.56	19.50	17.63	18.68
	16.25	14.92	14.63	15.31	15.87
	15.73	20.07	16.60	19.47	
	17.49	17.07	18.39	17.63	17.18
	16.89	14.72	15.04	16.58	16.04
**mean**	16.673	16.754	16.723	17.323	16.944
**95%CI**	14.846	14.444	14.731	15.421	14.886
	18.500	19.065	18.715	19.225	19.003
**SD**	1.741	2.201	1.898	1.532	1.294

Change in Ln total biovolume of objects over 10 cell size

day	T0	T5	T7	T10	T20
**NO**	17.01	-3.14	-0.58	1.65	0.57
	15.99	-0.09	-0.26	1.42	
	16.08	-1.94	-2.01	0.92	0.22
	16.65	-0.70	1.17	1.48	0.78
	17.63	-2.50	-2.48	-1.75	0.84
	15.22	-2.13	-16.04	2.70	
**mean**	16.431	-1.750	-3.367	1.070	0.603
**95%CI**	15.541	-2.950	-10.024	-0.505	0.157
	17.322	-0.550	3.291	2.645	1.048
**SD**	0.848	1.143	6.344	1.501	0.280
**Placebo**	14.26	0.94	1.91		
	19.42	-0.86	0.08	-1.79	-0.74
	16.25	-1.33	-1.61	-0.94	-0.37
	15.73	4.34	0.87	3.74	
	17.49	-0.42	0.91	0.14	-0.31
	16.89	-2.18	-1.85	-0.31	-0.85
**mean**	16.673	0.082	0.052	0.168	-0.568
**95%CI**	14.846	-2.362	-1.522	-2.470	-0.993
	18.500	2.526	1.625	2.806	-0.142
**SD**	1.741	2.329	1.499	2.124	0.268

S3b Summary tables for FEV1 individual patient data.

best FEV_1

day	T0	T7	T10	T20
NO	1.19	2.42	2.14	1.31
	0.78		0.74	0.75
	0.96	1.13	1.00	
	1.58	1.49	1.23	1.40
	2.53	2.84	2.51	3.10
	0.76	1.93	1.22	1.54
**mean**	1.300	1.962	1.473	1.620
**95%CI**	0.591	1.107	0.746	0.527
	2.009	2.817	2.201	2.713
**SD**	0.675	0.689	0.693	0.880
Placebo	1.01	1.27	1.22	1.02
	1.83	1.84	2.23	2.01
	1.46	1.91	1.77	1.76
	2.49	2.75	2.88	2.78
	1.16	1.34	1.36	1.32
	1.28	1.41	1.62	1.42
**mean**	1.538	1.753	1.847	1.72
**95%CI**	0.966	1.169	1.199	1.063
	2.110	2.337	2.494	2.374
**SD**	0.545	0.556	0.617	0.624

% predicted FEV_1

day	T0	T7	T10	T20
NO	37	75	67	41
	26		25	25
	34	39	34	
	52	49	41	46
	74	83	74	79
	18	47	30	37
**mean**	40.2	53.1	45.2	45.6
**95%CI**	19.017	25.947	23.727	20.495
	61.283	80.220	66.606	70.705
**SD**	20.138	21.855	20.430	20.219
Placebo	22	28	27	23
	52	53	64	58
	45	59	55	55
	77	86	87	87
	39	45	46	45
	39	43	49	43
**mean**	45.7	52.3	54.7	51.8
**95%CI**	26.480	31.815	33.650	29.617
	64.853	72.852	75.683	74.050
**SD**	18.283	19.552	20.027	21.170

Supplementary Fig S1. NO metabolite concentrations in blood plasma and erythrocytes for CF patients receiving either 10 ppm inhaled nitric oxide or placebo (means ± SEM) did not show statistically significant differences between groups. **A)** and **B)** plasma nitrite, n=6 per group; **C)** and **D)** plasma nitrate, n=6 per group; **E)** and **F)** total nitrosation products (RXNO), n=5 per group; **G)** nitrosylhemoglobin (NO-Heme), n=6 per group.

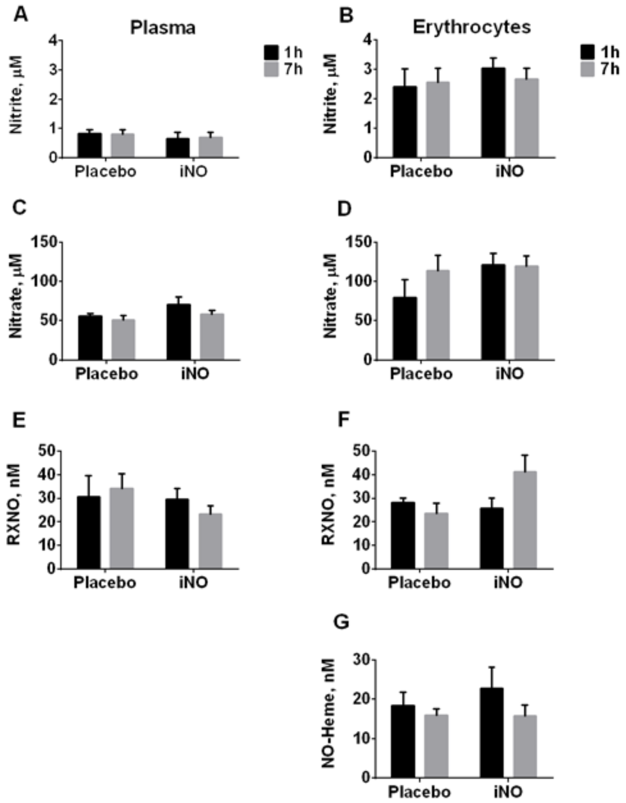

Supplementary Figure S2: NO does not affect FISH identification of *P. aeruginosa:* CSLM images showing that NO does not mediate fluorescence quenching or inhibition of FISH probe reporter. Images (both low and high magnification) show *P. aeruginosa*, following 24 h treatment with 450 nM NO (500 µM SNP) or HBSS (controls), as identified by species-specific FISH probe (green) and counter stained with propidium iodide (red) to identify total bacterial cell population. Merged images (right hand column) demonstrate clean overlay of both signals and therefore no effect of NO on FISH identification of *P. aeruginosa*. Scale bars: 10 µm.

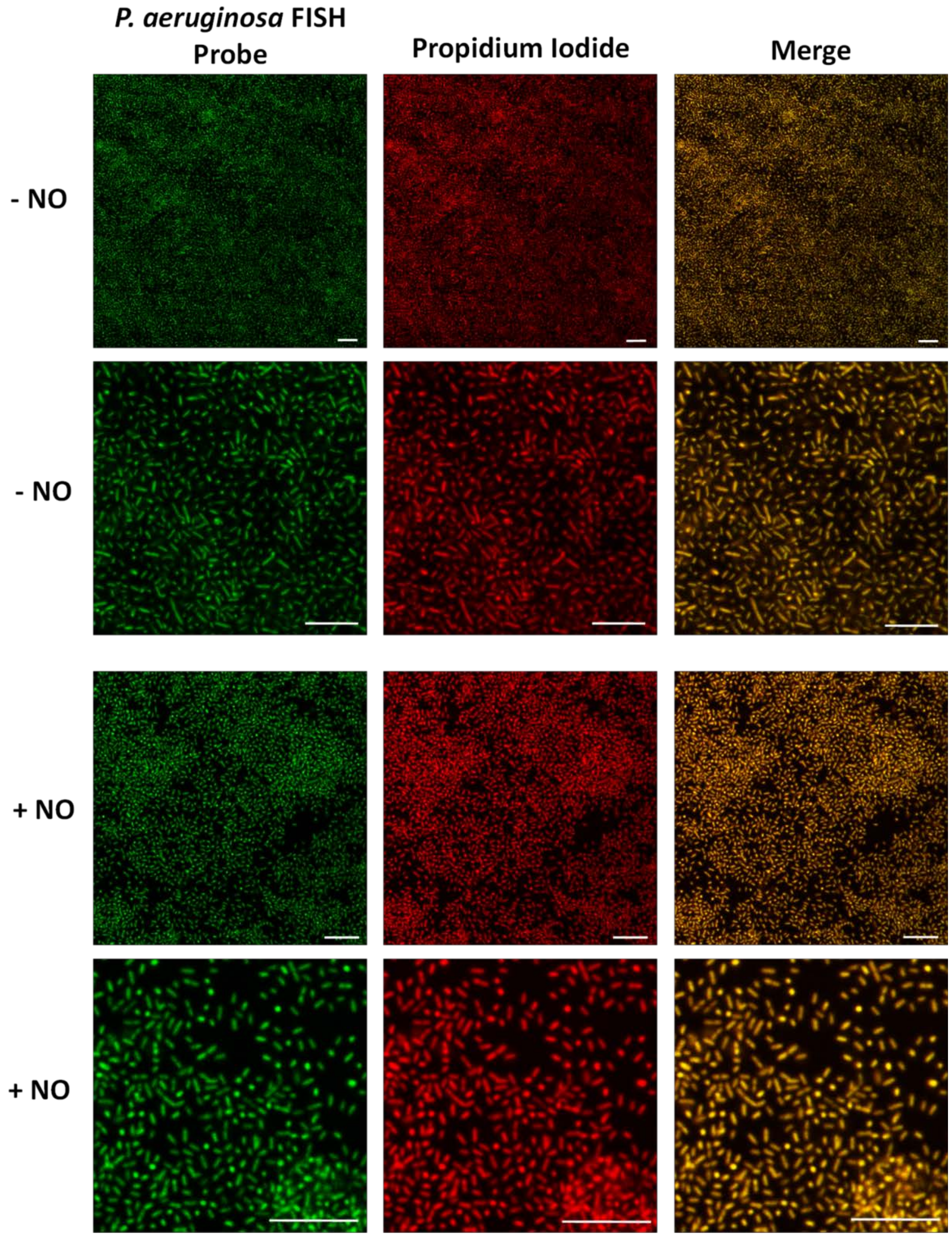

Supplement: Document S1. Figures S1 and S2 and Tables S1–S3 [file mmc1.pdf]
